# Editorial: Women in microbial physiology and metabolism: 2022

**DOI:** 10.3389/fmicb.2023.1268568

**Published:** 2023-08-14

**Authors:** Graciela L. Lorca, Sabine Kleinsteuber

**Affiliations:** ^1^Department of Microbiology and Cell Science, Genetics Institute, Institute of Food and Agricultural Sciences, University of Florida, Gainesville, FL, United States; ^2^Department of Environmental Microbiology, Helmholtz Centre for Environmental Research – UFZ, Leipzig, Germany

**Keywords:** biofilm, mycobacteria, host-pathogen interface, phyto-pathogen, antimicrobial resistance, methanogens, cyanobacteria, gut microbiota

There is still a gender imbalance in the field of STEM research. According to the UNESCO Institute for Statistics, only 31% of the world's researchers are women. They continue to be underrepresented in the highest academic positions, with only 32% of full professor positions in the U.S. held by women.^1^ As highlighted by UNESCO, science and gender equality are essential to ensure sustainable development. The present Research Topic highlights recent work performed by female researchers across the entire breadth of Microbial Physiology and Metabolism. It covers recent insights into the physiology of bacteria that are pathogenic to humans or plants, the prevalence of antimicrobial resistance genes in different environments, the molecular mechanisms of microbial light harvesting systems, and novel application potential of microbial metabolism in biotechnology.

## 1. Human-pathogenic bacteria

*Pseudomonas aeruginosa* is an opportunistic pathogen often associated with nosocomial infections. Its ability to form robust biofilms is related with enhanced virulence and antibiotic resistance. Bisht et al. studied temperature-specific adaptations relevant to both industrial/environmental and medical settings, as well as genetic requirements that affect biofilm formation in *P. aeruginosa*. It was found that temperature can affect production of biofilm through the expression of two previously uncharacterized genes, *PA14_50070* and *PA14_67750*, thereby affecting the matrix composition of the biofilm.

The availability of iron is involved in the regulation of the expression of bacterial pathogenicity determinants within the host. Islam et al. investigated the role of iron restriction on *Staphylococcus aureus*. It was found that low iron concentrations result in inhibition of aerobic respiration and the transient display of the small colony variant (SCV) phenotype. Prolonged iron starvation led to the emergence of amino-glycoside resistant SCVs, which potentially serve as a source of antibiotic resistance in iron-restricted host niches.

There is an urgent need to identify new antimicrobial compounds for antibiotic resistant bacteria. A well-defined target for the design of antimicrobials in *Mycobacterium tuberculosis* is the topoisomerase I (TopA). Garcia P. et al. investigated the binding site of the toxin MazF4 in *M. tuberculosis*. Using temperature-sensitive complementation experiments, carboxyterminal truncations and molecular simulations, the TopA C-terminal sequence motif was identified as the mechanistic target of the endogenous toxin MazF4.

## 2. Phyto-pathogenic bacteria

*Pectobacterium carotovorum* causes bacterial soft rot and is known to be more virulent under low oxygen concentrations (Babujee et al., 2012). Fekete et al. applied transcriptomics, proteomics, phenotypic assays, and inductively coupled plasma-mass spectrometry to elucidate the molecular regulation of oxygen sensing in this important plant pathogen. They found that the soluble globin-coupled sensor protein DgcO interacts with chemotaxis proteins, affects the expression of flagella genes, alters the cellular metal homeostasis, and plays a key role in transition between aerobic and anaerobic growth.

*Candidatus* Liberibacter asiaticus is one of causal agents of huanglongbing disease, which has caused devastation in the citrus industry worldwide. The lack of effective antimicrobials for the management of this disease is a direct consequence of the absence of methods to culture this microorganism in laboratory conditions. Using a variety of bacterial surrogate systems, Garcia L. et al. evaluated the role of a serralysin-like metalloprotease of *Can*. Liberibacter asiaticus on the modulation of components of the bacterial extracellular matrix. It was found that overexpression of Las1345 negatively impacted cell motility, exopolysaccharide production, and biofilm formation in *Xanthomonas campestris* pv. *campestris* (*Xcc*).

## 3. Antimicrobial resistance genes

Understanding the environmental fate of antimicrobial resistance genes (ARGs) is crucial within the One Health approach (McEwen and Collignon, 2018) and a prerequisite for more effective regulations on wastewater treatment and discharge. Haenelt et al. monitored the spread of sulfonamide resistance genes and class 1 integron ARG cassettes in a river ecosystem receiving effluents from wastewater treatment. They observed a decrease in ARG abundance with increasing distance from the wastewater discharge but an increasing relative abundance of class 1 integrons. Magnúsdóttir et al. surveyed public metagenome sequences from urban environments for the occurrence and phylogenetic distribution of different ARG classes and found that multidrug and glycopeptide ARGs are ubiquitous among urban bacteria.

## 4. Other topics

In contrast to bacteria and yeasts, archaea are uncommon in biotechnological applications, apart from the central role of methanogenic archaea in producing biogas from organic waste streams. However, this anaerobic digestion process relies on complex microbial consortia dominated by bacteria (De Vrieze and Verstraete, 2016), whereas pure culture applications of methanogens in industrial scale are restricted to the biomethanation of hydrogen in Power-to-Gas approaches (Logroño et al., 2023). Carr and Buan review the opportunities for archaeal bioproducts beyond methane and describe how the unique and highly efficient metabolism of methanogens can be harnessed for the bioproduction of terpenes and future applications making use of synthetic biology.

Elemene is a plant sesquiterpene with antiproliferative effects on cancer cells, but its use as an antitumor agent has been approved only in China. Although elemene seems to be promising as a chemotherapeutic agent (Jiang et al., 2017), there are still knowledge gaps that hamper its general application. In a preliminary study with rats, Gu et al. observed that intraperitoneal elemene injection modulates the gut microbiome and fecal metabolites, but further studies are needed to understand the physiological effects of elemene on gut microbiota.

Cyanobacteria such as *Synechococcus* contribute to oxygen release in the atmosphere by photosynthesis using phycobilisomes. These complex structures are composed of an allophycocyanin core surrounded by phycocyanin and two phycoerythrin (PE) types: PEI and PEII. Carrigee et al. found that MpeV in *Synechococcus* sp. WH8020 showed differential enzymatic capabilities by having lyase-isomerase activity on the PEII β-subunit but only lyase activity on the PEI β-subunit. Though structural modeling and site directed mutagenesis, the authors show that the phycoerythrobilin isomerization activity of MpeV is modulated depending on the amino acid that is located at position 141 within its phycoerythrin-I β-subunit substrate. These results in combination with structural models led the authors to propose that identity of the residue at this position in the β-subunits may be used to predict which phycobilin is bound on both PEI and PEII.

## Author contributions

SK: Writing—original draft, Writing—review and editing. GL: Writing—original draft, Writing—review and editing.

## Footnotes

^1^ https://www.aauw.org/resources/research/barrier-bias/
